# Effects of a Low-Dose Spirulina/Turmeric Supplement on Cardiometabolic and Antioxidant Serum Markers of Patients With Abdominal Obesity

**DOI:** 10.3389/fnut.2020.00065

**Published:** 2020-05-19

**Authors:** Arlene Gómez-Téllez, Diego Sierra-Puente, Regina Muñoz-Gómez, Amelia Ibarra-Pitts, Martha Guevara-Cruz, Marcela Hernández-Ortega, Gabriela Gutiérrez-Salmeán

**Affiliations:** ^1^Facultad de Ciencias de la Salud, Universidad del Valle de Toluca, Mexico City, México; ^2^Centro de Investigación en Ciencias de la Salud, Facultad de Ciencias de la Salud, Universidad Anáhuac México, Mexico City, México; ^3^Departamento de Fisiología de la Nutrición, Instituto Nacional de Ciencias Médicas y Nutrición Salvador Zubirán, Mexico City, México

**Keywords:** abdominal obesity, antioxidants, cardiometabolic, nutraceutics, spirulina, turmeric

## Abstract

Obesity is one of the greatest public health problems worldwide. It is associated with underlying low-grade inflammation, thus is a risk factor for the development of cardiometabolic alterations. Functional foods, such as spirulina and turmeric, in the form of nutraceutics have been considered to exert not only an antioxidant effect but also modulate mechanisms in the metabolic pathways underlying cardiometabolic disruptions. We aimed to study the effectiveness of supplementation with a *Spirulina maxima*/*Turmeric longa* mixture (266 mg/156.6 mg) on body composition, lipemic, and antioxidant status in patients with abdominal obesity. To achieve this, 43 patients were included (control group, *n* = 21, and experimental, *n* = 22), in a double-blind randomized controlled trial. Both groups were daily supplemented, orally, for 12 weeks. After 3-month supplementation (altogether with individualized dietary management), both groups showed a decrease in body weight, fat mass, and abdominal circumference; however, no intergroup statistical differences were found. The same phenomenon was observed concerning biochemical metabolic markers; nevertheless, an obvious trend favoring spirulina/turmeric supplementation can be appreciated. Finally, both groups significantly increased their serum antioxidant status, although the supplemented groups showed a two-fold accrue vs. placebo.

## Introduction

It is well-known that obesity is one of the greatest public health problems worldwide. It is associated with underlying low-grade inflammation, thus is a risk factor for the development of cardiometabolic alterations such as type 2 diabetes, dyslipidemia, hypertension, fatty liver disease, and some cancers (1, 2). Mexico is considered among the first places in global obesity, with a combined prevalence of overweight and obesity (O/O) of 72.5%. For its side, the prevalence of abdominal obesity reaches 76.6%, according to the Half Way National Survey of Health and Nutrition (ENSANUT MC) 2016 (3).

The metabolic syndrome is characterized by a specific phenotype of abdominal obesity associated with a set of metabolic aberrancies, including hypertension, insulin resistance associated with hyperinsulinemia, glucose intolerance, and dyslipidemia, which is characterized by hypertriglyceridemia and a low serum concentration of HDL-C (2, 4). In addition, the inefficient management of caloric excess by visceral fatty tissue causes hypertrophy of the adipocyte, making it a proinflammatory cell (5). All together, these pathological conditions ground medical complications that not only lead to great morbidity and mortality but also reduce the quality of life.

Although obesity and its associated cardiometabolic alterations, including the proinflammatory context, have a complex etiology and risk factors include age, gender, and family pathological history, it has been well-documented that unhealthy lifestyles (sedentary lifestyle, obesity, poor eating habits, smoking) are also important modulators of their progression (6, 7).

Hence, lifestyle changes—including dietary interventions, increased physical activity, and behavior counseling modification—to promote weight are the cornerstone treatment. Nevertheless, the use of concomitant functional foods has been of growing interest. Functional foods were defined by the International Life Sciences Institute (ILSI) Europe as those foods that satisfactorily demonstrate the beneficial effect on functions of the organism, in addition to their intrinsic nutritional effects, in such a way that they are appropriate to improve the state of health and well-being, reduce the risk of disease, or both (8). Among such, there are turmeric and microalgae.

Turmeric (*Turmeric longa*) is a traditional rhizome, originally from China and Southeast Asian regions. Curcuminoids, the yellow pigments in turmeric, have been identified as the most bioactive principles and were characterized as a group of bis-α,β-unsaturated β-diketone polyphenols; namely, curcumin, demethoxycurcumin (DMC), and bis-demethoxycurcumin (BDMC). Commercially available turmeric is a mixture of those three curcuminoids: curcumin (72–78%), DMC (12–18%), and BDMC (3–8%), and has been widely used as dietary supplement (9). Several studies show that the bioactive compounds of turmeric participate in the control of inflammation, cell growth, and apoptosis, in brief, through their antioxidant action. This may be mediated by blocking tumor necrosis factor alpha (TNFα). It can also attenuate the generation of free radicals and reduce the release of interleukins through the modulation of nuclear factor-kappa beta (NF-κB) (8). The dose required to achieve the mentioned beneficial effects may vary, depending on existing disease conditions. For example, in cancer, curcumin may require higher concentrations (1.5–4 g/day) compared to inflammatory conditions, which need <0.5 g of curcumin (10). A study indicate that a consumption of 2.8 servings per month (50 mg/day) of turmeric like curry is related to a reduction in plasma levels of both glucose and triglycerides in patients presenting overweight and altered lipid and glucose concentrations (10). Moreover, studies in rats, with lipid alterations, showed that the administration of curcumin over 8 weeks decreased plasma concentrations of both cholesterol and triglycerides (11). A study conducted in mice treated with 0.02% w/w curcumin after 18 weeks proved significant lower levels of plasma CT, LDL-C, apoB, and TG, similar to the cholesterol-lowering effect of lovastatin (12). The 6-week administration of curcumin to rabbits fed a high cholesterol diet increased plasma HDL-C and decreased plasma LDL-C, lipoprotein A, hepatic cholesterol, and hepatic apoB mRNA compared to placebo (13). However, the average consumption within populations that, in fact, ingest a relatively high amount of turmeric reaches up to 60–100 mg of curcumin.

For its side, Spirulina (*Arthrospira*) refers to filamentous microalgae, belonging to the class of cyanobacteria with the characteristic of photosynthetic capacity. It naturally grows in alkaline water reservoirs with high salt content, in subtropical waters and tropical areas including America, Asia, and Central Africa. Three species of spirulina (*Spirulina platensis, Spirulina maxima*, and *Spirulina fusiformis*) are the most studied for their high nutritional content with therapeutic values (14). The nutritional qualities include a high protein content (60–71% depending on the strain), phenolic acids, tocopherols, carotenes, and linolenic acid. Spirulina lacks cellulose cell walls; therefore, it does not require chemical or physical processing to be digestible (15). Studies have been conducted to evaluate the therapeutic benefits in a variety of diseases including hypercholesterolemia, hyperglycemia, cardiovascular diseases, inflammatory diseases, cancer, and viral infections. The cardiovascular benefits of spirulina are lipid lowering, antioxidant, and anti-inflammatory (15). These effects are achieved by means of different doses that varies from 1 to 10 g per day (16). Spirulina produces two phycobiliproteins: C-phycocyanin (C-PC) as the major pigment and allophycocyanin (APC), which is present in much smaller quantities, approximately at a 10:1 ratio. The C-phycocyanin level varies based on growing conditions and may constitute up to 20% of the dry weight of Spirulina (17). Phycocyanin [PC; (18)] is one of the main pigments of Spirulina, eliminates reactive oxygen and nitrogen species (ROS and RNS, respectively), and prevents oxidative damage, which may explain its beneficial effects (15). After 3 months of supplementation with 2 g of spirulina to patients with obesity and hypertension, a significant reducing effect on LDL-C showed, and the concentration of IL-6 considerably improved the total antioxidant status and insulin sensitivity ratio (19).

Considering the beneficial aspects of both turmeric and spirulina, it is proposed that the maximum spirulina/turmeric longa mixture be used to make a synergy and increase its lipid lowering, antioxidant, and anti-inflammatory actions.

## Materials and Methods

The procedures were followed in accordance with the ethical standards of the responsible committee on human experimentation. The study was approved by the Ethics and Research Committee, national registration number CONBIOETICA-15-CEI-004-201804 and was conducted following international guidelines (e.g., Helsinki Declaration) and national regulations (e.g., NOM-012-SSA3-2012).

Patients were recruited from private practice offices and invited to participate in the study. Inclusion criteria were both sexes, between 18 and 60 years of age, with abdominal circumference >80 cm (women) or 90 cm (men). After signing the informed consent, patients were randomly allocated into one of two double-blinded groups: control or experimental. The first group received placebo (maltodextrin, 500 mg) and the second one *Spirulina maxima*/*Turmeric longa* (266 mg/156.6 mg, respectively), both orally, in fasting conditions, every 24 h for 12 weeks. The mixture was designed taking as a reference the minimum concentrations at which spirulina and turmeric generate a beneficial effect: 1 g of spirulina and 0.5 g of turmeric. The proportion of these was adjusted to the 500 mg that the capsule should contain so it was dosed in the same amount as the placebo. It was decided that the lowest doses of these functional foods be used since there are different studies that prove the pro-oxidant effect that high concentrations of these foods can generate (20, 21).

Anthropometric measurements (weight, height, waist circumference, body fat percentage) and blood pressure were registered, and further, the body mass index (BMI) was calculated. A blood sample was collected, through a venous puncture in the internal ulnar vein, using the vacutainer system, to determine the levels of glucose, triglycerides, uric acid, antioxidants, and curcumin in serum.

Glycemia, triglycerides, and serum uric acid were assessed with commercially (RANDOX México) available kits and enzymatic–spectrophotometric techniques.

For total antioxidant status, the Randox procedure was used to determine the total antioxidant status (TAS) in serum. TAS was measured in the system that generates the ABTS® cation radical (HX-FeIII + H_2_O_2_), with absorbance at 600 nm. In the event of the presence of antioxidants in plasma, absorbance is diminished. A synthetic antioxidant (6-hydroxy-2,5,7,8-tetramethylchroman-2-carboxylic acid) was used as the standard (22).

For curcumin determination, 100 μl of serum was mixed with 300 μl of methanol. The mix was vortexed for 1 min and incubated at 75°C for 10 min. When the time was finalized, the mixture was vortexed for 1 min and then centrifuged for 3 min at 5,000 rpm. The supernatant was separated and used for curcumin determination by the Folin–Ciocalteu method. For this purpose, 10 μl of the supernatant was mixed with 600 μl of water and 50 μl of Folin reagent. The mixture was vortexed for 1 min and then mixed with 150 μl of sodium carbonate at 20% and 190 μl of water. The mixture was vortexed for a minute and allowed to stand for 2 h at room temperature. Then 300 μl was placed on a microplate and read at 760 nm. A standard curve of curcumin was used for calculation (23).

For measuring the weight and body fat percentage, a bioelectric impedance scale was used; for the height, a wall stadiometer; for the waist circumference, a metal tape; for collecting the blood pressure, a digital arm baumanometer; and finally, the set of blood samples was performed by spectrophotometry.

An individualized eating plan also was prescribed to each patient, and the energy requirement was calculated according to the Mifflin–St Jeor equation (24).

Patients were monitored twice again (at 4 and 8 weeks), measuring the anthropometric features and blood pressure, as well as adherence to the diet by means of a short-term reminder. At the end of the period, the blood sample, anthropometric variables, and blood pressure were obtained again.

### Statistical Analysis

Basal characteristics are expressed as mean ± standard deviation, for continuous variables; qualitative data are expressed as frequencies.

After analyzing the data distribution results (Kolmogorov–Smirnov test), Student's paired *t*-test was used to assess intragroup changes, while independent *t*-tests were used to compare the effects between groups. Significance was considered when *p* < 0.05. SPSS version 25 and GraphPad Prism version 5 were used as statistics software.

## Results

**Figure 1:**
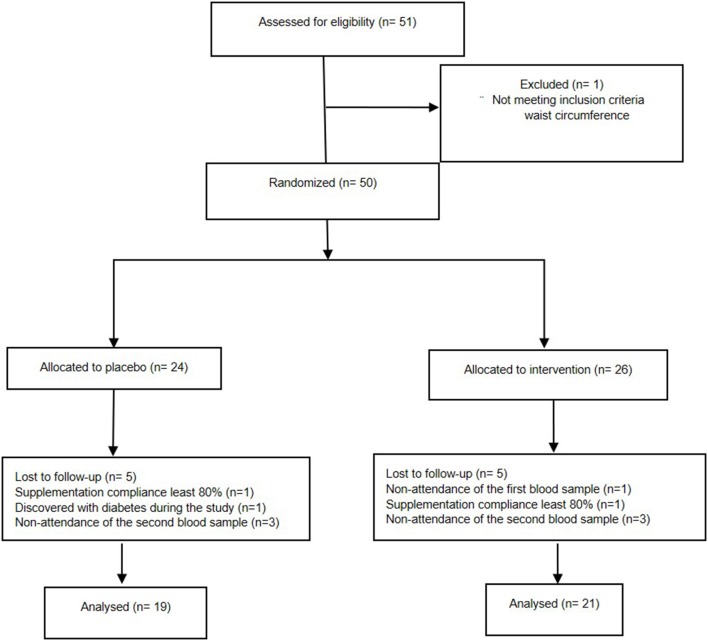
CONSORT flow diagram.

A total of 51 subjects were screened for eligibility and further 50 were randomized; one patient was eliminated because no first blood sample could be recollected, six patients were eliminated because no second blood sample could be recollected; one patient was eliminated because diabetes was discovered during the study; and two patients were eliminated because they did not meet the supplement minimum compliance. Finally, 40 patients were included in the analysis (control group, *n* = 19, and experimental, *n* = 21; Figure 1).

The overall rate of supplementation compliance (i.e., consuming at least 80% of the capsules) was 95.3%; one enrolled participant of each groups was eliminated from analysis due to poor compliance. This per protocol analysis yielded a final study power of 0.84 (for a one-sided test with a confidence of 95%), considering abdominal circumference as the outcome variable—due to it being a selection criteria and its role on cardiometabolic disruptions and antioxidant status, as stated in the background section.

Participants' (13 males, 27 females) mean age was 35.50 ± 10.65 years. Weight mean was 78.25 ± 14.02 kg yielding an average overweight BMI (29.30 ± 4.14 kg/m^2^); however, fat mass mean value (36.20 ± 6.27%) reveals an obese sample in which central adiposity (mean waist circumference 95.55 ± 10.52) prevails. Both systolic and diastolic arterial pressures were found to be within normal ranges (114.0 ± 11.60 and 73.0 ± 8.57 mmHg, respectively). Biochemical markers were, in average, not altered: triglyceridemia 122.6 ± 72.54 mg/dl, glycemia 82.40 ± 18.22, uric acid 4.60 ± 2.68; however, their standard deviations suggest that some of the participants did actually have serum metabolic derangements.

As shown in Table 1, at baseline, there were no significant differences between groups in their general characteristics.

**Table 1:** Baseline cardiometabolic profile.

**Parameters**	**Placebo (*n* = 19)**	**Supplement (*n* = 21)**
Weight (kg)	78.20 ± 15.42	78.30 ± 13.12
BMI (kg/m^2^)	28.70 ± 5.0	30.0 ± 3.3
Body fat (%)	37.20 ± 6.63	35.80 ± 6.08
Waist circumference (cm)	93.90 ± 11.15	97.90 ± 10.04
Systolic blood pressure (mmHg)	112.5 ± 10.54	116.0 ± 12.52
Diastolic blood pressure (mmHg)	70.50 ± 7.77	77.00 ± 9.22
Triglyceridemia (mg/dl)	128.4 ± 55.91	116.7 ± 85.98
Glycemia (mg/dl)	80.60 ± 12.44	86.60 ± 21.65
Serum uric acid (mg/dl)	4.40 ± 1.20	4.70 ± 3.51
Total antioxidant status (mmol/L)	2.40 ± 1.14	2.20 ± 0.66

**Figure 2:**
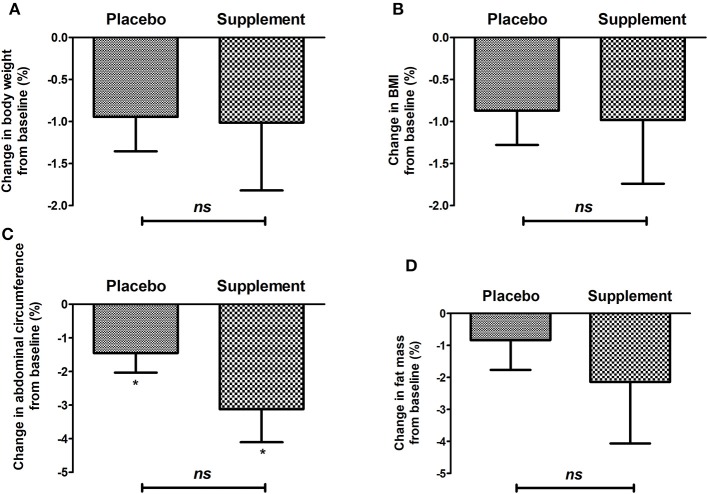
Change in anthropometric variables: **(A)** body weight, **(B)** BMI, **(C)** waist circumference, **(D)** body fat of the placebo group and intervention after 12 weeks of treatment. *p < 0.05 after paired *t*-test; *ns*, no significant difference after Mann–Whitney *U*-test.

Figure 2 shows that after a 3-month supplementation (altogether with individualized dietary management), both groups showed a decrease in body weight (−0.94 ± 1.78 vs. −1.13 ± 4.03, for control and supplement, respectively), BMI (−0.86 ± 1.78 vs. −0.91 ± 3.62), fat mass (−0.30 ± 4.47 vs. −2.19 ± 9.24), and abdominal circumference (−1.45 ± 2.47 vs. −3.21 ± 4.72); later, a significant reduction when compared from baseline was found in both groups; however, no intergroup statistical differences were found. The same phenomenon was observed for DBP (7.44 ± 12.96 vs. 3.28 ± 12.28), while a significant difference was found in SBP from baseline (−4.23 ± 9.39 vs. −4.20 ± 10.62).

**Figure 3:**
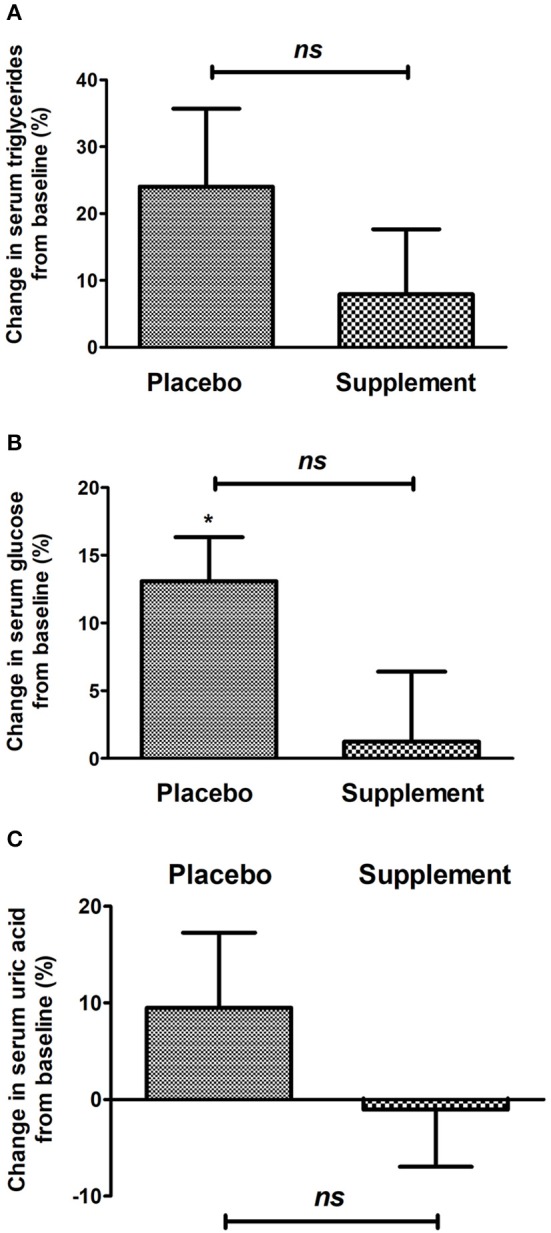
Change in biochemical variables: **(A)** triglyceridemia, **(B)** glycemia, **(C)** serum uric acid of the placebo group and intervention after 12 weeks of treatment. *p < 0.05 after paired *t*-test; *ns*, no significant difference after Mann–Whitney *U*-test.

For its side, Figure 3 shows the percentual change in biochemical metabolic markers. Between groups, no significant difference was found; nevertheless, an obvious trend favoring curcuma/Spirulina supplementation can be appreciated as serum not glucose, triglycerides not uric acid virtually changed.

We also analyzed the compliance to nutrition counseling: disappointingly, we found that only 31.25% of the control and 22.22% of the supplemented patients concurred with their total energy needs (90–110% of adequacy). None of the participants covered the international physical activity recommendations (i.e., at least 150 min/week of moderate–vigorous exercise).

**Figure 4:**
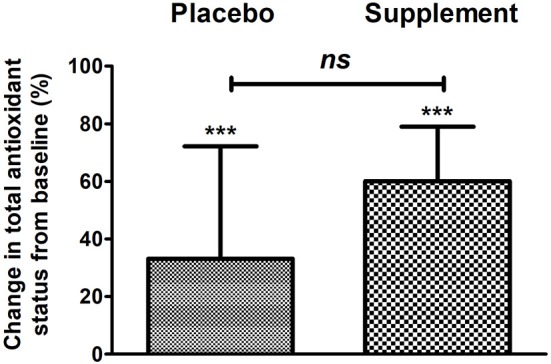
Change in serum antioxidant status of the placebo group and intervention after 12 weeks of treatment. ***p < 0.001 after paired *t*-test; *ns*, no significant difference after Mann–Whitney *U*-test.

**Figure 5:**
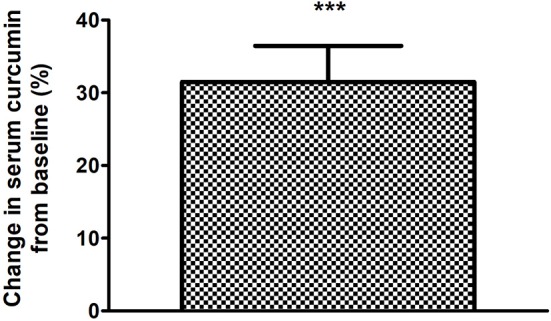
Change in serum curcumin of the supplemented group and intervention after 12 weeks of treatment. ***p < 0.001 after paired *t*-test.

Finally, total antioxidant status in the blood significantly increased in both groups, although no difference was found between them when comparing between groups; however, a greater effect is seen in the intervention group, as seen in Figure 4. Serum curcumin significantly increased after 12 weeks of supplement consumption as it can be appreciated in Figure 5.

## Discussion

Obesity and its metabolic complications have increased in recent decades, becoming a serious global health problem (25). Obesity is related to low grade chronic inflammation and is a risk factor for cardiovascular diseases (2, 26). Consider that functional foods have benefits beyond nutritional aspects and can play a role in reducing the risk of certain diseases (27). The development of strategies related to bioactive food compounds to prevent or mitigate the consequences related to obesity is of great interest (28, 29). So far, the effects of spirulina and turmeric have been studied separately or in combination with other foods. In this study, the effect of supplementation with a mixture of *Spirulina maxima/Turmeric longa* on body mass, body mass index, fat mass, made changes between the placebo and intervention group, but were not significant; however, a greater decrease in body fat is observed graphically in the intervention group. Abdominal circumference and systolic blood pressure had a significant reduction when compared from baseline in both groups; however, no intergroup statistical differences were found. Changes in serum glucose levels, triglycerides, and uric acid, of the participants, were not significant between the placebo and intervention group; nevertheless, an obvious trend favoring *Spirulina maxima/Turmeric longa* supplementation can be observed. Unfortunately, both groups do not show adherence to the individualized eating. None of the participants covered the international physical activity recommendation. The effect observed in the intervention group is clinically lowered cardiometabolic risk, thereby reducing health care costs, increasing the quality of life of patients, and having alternatives that benefit health.

The results of a study conducted in Mexico showed that maximum spirulina has lipid lowering effects, especially in the concentration of TG and LDL-C and positive effects on lowering blood pressure (30, 31). Studies have demonstrated the effect of spirulina supplements on the lipid profile in patients with type II diabetes mellitus (DM2), reporting a significant reduction in TG levels after the intervention (32). The results of a meta-analysis of controlled clinical trials revealed a significant effect of spirulina supplementation in reducing plasma concentrations of total cholesterol, LDL-C, and triglycerides, and increasing those of HDL-C (33). In the study by Szulinska et al., a significant decrease in body mass, BMI, waist circumference, and glucose was found, after supplementation of 2 g of spirulina maximum for 3 months in patients with obesity (19). One study reviewed the effect of *Spirulina maxima* supplementation in combination with a systematic program of physical exercise, observing improvement on BMI and blood lipid profile in overweight and obese men, mainly in individuals with dyslipidemia (33).

Otherwise, curcumin has beneficial effects on the state of various diseases involving chronic inflammation (34). Most of these benefits are due to its antioxidant and anti-inflammatory effects (35, 36). Studies have shown the effect of turmeric on the decrease in glucose, cholesterol, and triglycerides in the blood (9–11). We also obtained this effect in the intervention group. One study evaluated a high-fat diet in animal models and reported that ingestion of curcumin inhibited weight gain, reduced fat accumulation, and significantly improved serum lipid profile (including serum triglyceride levels, total cholesterol, LDL cholesterol, HDL cholesterol, and free fatty acids) (37). In a randomized double-blind clinical trial in subjects with metabolic syndrome who received 1 g of curcumin with 5 mg of piperine for 8 weeks, short-term supplementation with the curcuminoid–piperine combination significantly improved oxidative and inflammatory status in patients with metabolic syndrome (38). We also obtained this effect in the intervention group, an obvious trend favoring *Spirulina maxima/Turmeric longa* supplementation in total antioxidant status. Studies that treat with curcumin can help in the management of oxidative and inflammatory conditions, metabolic syndrome, arthritis, anxiety, and hyperlipidemia (39).

Finally, although there is a clear-cut tendency in the supplemented individual toward an effect of greater magnitude, we believe no significant intergroup differences were found due to the fact that all cardiometabolic values (abdominal circumference, biochemical markers) have large variations, and in spite of the study power of 0.84, the sample size was simply not enough for normalizing standard deviations. Moreover, our inclusion criteria were rather lax (as individuals are within the real clinical scenario), and we did not screen for specific ranges of cardiometabolic values; hence, as stated above, large variations are expected. Ultimately, we do believe these are promising results since the phytochemical effect is always of a fair-moderate magnitude (compared to drugs) and, more importantly, a coadjuvant strategy, not an isolated solution nor curative agent, that should be consumed for long periods—therefore, maybe more supplementation time could increase the differences herein seen.

## Conclusions

The effect of supplementation with a mixture of *Spirulina maxima/Turmeric longa* on body mass, body mass index, fat mass, made changes between the placebo and intervention group, but were not significant; however, a greater decrease in body fat is observed graphically in the intervention group. For the abdominal circumference and systolic blood pressure, there was a significant reduction when compared from baseline in both groups; however, no intergroup statistical differences were found. A trend is observed that favors the intervention group in the decrease of glucose, triglycerides, and uric acid. The effect observed in the intervention group is clinically lowered cardiometabolic risk, thereby reducing health care costs, increasing the quality of life of patients, and having alternatives that benefit health.

## Data Availability Statement

The datasets generated for this study are available on request to the corresponding author.

## Ethics Statement

The studies involving human participants were reviewed and approved by IRB (Comité de Ética e Investigación de la Facultad de Ciencias de la Salud de la Universidad Anáhuac México) and registered as CONBIOETICA-15-CEI-004-201804. The patients/participants provided their written informed consent to participate in this study.

## Author Contributions

AG-T, MH-O, and GG-S designed the study, performed statistical analysis, drafted, wrote, and approved the final manuscript. DS-P, RM-G, and AI-P contributed to participant recruitment and treatment, drafting, and writing of the manuscript. MG-C collaborated with the statistical analysis and writing of the manuscript. All authors approved the final version of the manuscript.

## Conflict of Interest

The authors declare that the research was conducted in the absence of any commercial or financial relationships that could be construed as a potential conflict of interest.
